# Editorial Department

**Published:** 1888-10

**Authors:** 


### EDITORIAL DEPARTMENT.

### CONGRESS FOR THE STUDY OF TUBERCULOSIS IN MAN AND IN ANIMALS.

This Congress marks an era in the annals of veterinary medicine. It was called by some of the most eminent of scientific men to consider the most important question in disease that the physician has to contend with to-day—a disease which kills yearly in the United States a number of people equal to five times the Regular Army of the country. The most distinguished of physicians called a veternarian to preside over them in recognition of his peerage in science and his yeomanry in medical investigation.

The Congress met in the school of medicine at Paris, France, on July 25th to 30th. In the absence of M. Chauveau, the meeting was opened by M. Villemin, assisted by the Committee of Organization, MM. Butel, Cornil, Leblanc, Nocard, Rossignol, Verneuil and Petit.

M. Chauveau, Inspector-General of the veterinary schools of France, the anatomist, was elected President; MM. Villemin and Verneuil, Vice-Presidents; M. Petit, General Secretary; MM. Leclainche and Villemin, Jr., Cagny, Gallois, Piot and Thoinot, Secretaries.

The following were appointed Honorary Presidents:

*France.*—MM. Pasteur, Charcot, Hérard, Cornil, Arloing, Laulanie, Nocard.

*Hungary.*—Azary, Hutyza.

*Roumania.*—Babès, Kalindéro, Petresco.

*Denmark.*—Bang, Escherning.

*Italy.*—Branchi, Perroncito.

*Greece.*—Boussakis, Orphanides.

*England.*—Coats-Shingleton, Smith.

*Norway.*—Dahl.

*Brazil.*—De Saboia.

*Belgium.*—Degive, Laho, Malvoz, Van Hortsen.
*Spain.*—Espina y Capo, Vargas.
*United States.*—A. Jacobi, Page.
*Holland.*--Yanné, Thomassen, Van der Huys.
*Turkey.*—Zocros Pacha.
*Luxembourg.*—Siegen.

M. Chauveau opened the meeting by an eloquent discourse, in which he traced the importance of studies in relation to tuberculosis, and the history of the successive discoveries concerning the nature of the disease since Villemin. He recalled the experiments in the transmission of tuberculosis, the researches of Toussaint foreseeing the agent of the contagion ; then, apropos of the discovery of *Robert Koch*, he evoked the memory of Davaine : "It is under the name of the *bacillus of Koch*, that by an unconscious but unanimous agreement, we are pleased to designate the infectious agent of tuberculosis, a small attention to a great merit. Yes, without doubt, a small attention. We would like, however to see the practice generalized. For instance, ought not we to say the *bacillus of Davaine*, for that infectious agent of anthrax, the first virulent microbe which has been signaled, described and pictured, known under the name of *bacteridia of anthrax*. * It is this discovery which marks the debut of the period so fertile which we cross to-day.

"To such a work, so absolutely original, a point of departure in the march forward of all the infectious microbiology is attached a glorious honor."

Showing further the inherent difficulties in general biology, difficulties still greater in pathology, the orator expressed in his peroration the hopes of the future : "But the clouds will disappear. See what has become of those which hid the nature of the infectious diseases, since the works of our admirable Pasteur on the ferments have permitted the definite introduction in pathogeny of the role of the infinite entities. Yes, let us make light, and more light !

---

* One of the editors of this journal has always called this bacillus the "bacillus of Davaine," in his lectures since 1884.

Who knows? The pathologist will arrive some day, perhaps, in making his work as easy as that of the naturalist, as precise as that of the physician, as fertile in laws rigorously deducted as that of the mathematician. It will be the moment for our great-grandchildren to throw themselves into the abstract, and to put in algebraic formula the play of the cells as has been done for atoms.

"In waiting let us be useful. It is a noble aim to devote ones self to work. That which to the present has been consecrated to tuberculosis has been particularly fertile. Experimental and comparative pathology will continue the work. If it succeeds in establishing laws for confining the ravages of this terrible scourge; if it succeeds thus in augmenting the sum of life and of happiness on earth, it will have rendered to the individual, as to society, a signal service."

The address of M. Chauveau was followed by that of Prof. Verneuil. He noted the apparent small limit of a single subject, but added that the importance compensated for it. He recalled the medical work of Bouley and Bronerdel, veternarians, on glanders and hydrophobia. "Human and animal pathology are continually borrowing from each other; the veternarians take from the physicians their nosological forms, their general description, their great laws; the physician demands from the veternarian the result of their vivisections and of their experimental research on the transmission of the infectious diseases.

"Comparative pathology, finally, is a common field which you have seen, in the last few years, cultivated so brilliantly by Bouley and Vulpeau, but still indeed it is to-day for the first time that the physician and veterinarian unite, not singly but in numbers, and combine their efforts to study the numerous diseases which have the fatal power of decimating at the same time man and his servants—of being transmitted without pity from the one to the other.

"Gentlemen, if I am not mistaken, to-day is a great one. You will remember it, for to-day, in France, at Paris, in the modern Babylon, we affirm, with a loud voice, the unity of medical science, we proclaim the equality of those who cultivate it, and we demonstrate the fraternity which reigns among all its representatives."

The work of the Congress was then inaugurated by the two following communications :

*M. Cornil. Tuberculosis of the Mucous Membranes.*— "Guinea pigs which had swallowed a few drops of a culture of tubercles showed visible tubercles in the intestine from the fifteenth day after ingestion. In the mesenteric ganglia were found accumulations of cells after the fourth day, and distinct tubercles after the sixth.

"I have introduced into the vagina of several female guinea pigs, with every precaution to prevent lacerating the membrane, a few drops of a culture of the bacillus of tuberculosis. These pigs were killed the sixth, twelfth, twentieth and thirty-second day after the operation, and the following lesions were found : The uterine lesions were found at the os uteri ; they consist at first of a catarrhal condition of the mucous membrane containing some bacilli ; about the fifteenth day, small tuberculous follicles developed under the epithelium, which remain intact ; these tubercles are very distinct about the thirtieth day. These experiments demonstrate the possibility of a tuberculization of the genital mucous membranes without lesions of it, they allow us to believe in a tuberculous infection of the uterus by way of the vagina.

*M. Nocard.—The Danger of Tuberculous Meat and Milk. Means to Prevent it.*—"As far as milk is concerned every one agrees. The milk is not virulent except when the mammary gland is tuberculous, but the diagnosis of this localization is difficult, and often impossible, one must treat all tuberculous cows as if the gland was always invaded.

"Sale of milk from tuberculous cows should always be interdicted, and the milk from doubtful cows should be boiled. If uncooked milk is necessary in some cases, goats milk can be employed with perfect safety.

For meat, the question is a more complex one. From the works of Toussaint it would seem that tuberculosis is a general disease, *totius substantiœ*, and M. Bouely, adopting this belief, made a vigorous campaign in favor of *total*

*seizure*, which he succeeded in having adopted by the International Veterinary Congress in Brussels, in 1883. Since then numerous experiments have shown what conclusions of Toussaint were too absolute; it is established for instance, that in the immense majority of cases the virulence remains localized in the tubercular lesions only; that the blood and the muscular juice possess it only rarely.

These new ideas prevailed at the Veterinary Congress at Paris, in 1885, where M. Arloing had adopted the principle of *partial seizure* in all cases of localized tuberculosis. The Committee of Epizootic has adopted the following project for a decree:

"Meat coming from tuberculous animals are excluded from consumation.

1st "If the lesions are generalized, that is not confined exclusively to the visceral organs and their lymphatic gangalia:

2d. "If the lesions, though localized, have invaded the greater part of a viscera, or show themselves by an eruption on the walls of the chest, or of the abdominal cavity."

Of forty Guinea pigs inoculated with the juice of meat coming from ten cows, tuberculous to the last degree, a single one only, succumbed to tuberculosis, fifty-nine days after the inoculation. In a first series of experiments, on eleven cows, no possible result was obtained. * * * In conclusion, "the meat of tuberculous animals can, in certain cases, offer some danger, but these dangers are exceptional, and little to be feared."

Meeting of July 26th, M. Chauveau presiding.

M. Arloing proposed the following conclusions:

1st. Immediate inscription of tuberculosis in the list of of contagious diseases designated in the law of 1881.

2d. Absolute prohibition of the meat of tuberculous animals, until there shall have been found a means to render it inoffensive.

M. Arloing continued with a resumé of the modus of obtaining the proper legislation and of aiding inspection.

M. Nocard obtained but one positive result in forty experiments. He and M. Chauveau had obtained positive results in inoculation 1 in 2 ; M. Galter 5 in 22.

M. Bang, (Copenhagen) said that the microscopic examination of milk in tuberculous cows is always indicated. In 21 cases, where the udder did not seem affected, he found the milk virulent in two cases. If the milk is not always virulent, it is always suspicious. In Denmark the dairies are inspected every two weeks for tuberculosis.

A temperature of 85° C., is necessary to destroy the virulence of tuberculous milk ; but at a temperature of 60° to 70° this virulence is so attenuated that infection by the digestive tract is not to be feared.

M. Baillet, (Bordeaux), said that his conclusions approach those of M. Nocard, rather than those of M. Arloing, and proposed more moderate measures than those of the latter.

M. Butel, (of Meaux), said that it was not a question of commercial interest, but of public hygiene. The interest of the farmer could be protected by indemnity—sanitary police could not be carried out without money.

MM. Grissonache, Veyssiére, Spellman, Rossignol and Guirand reported the prevalence of tuberculosis, and substantiated its contagion from one animal to another.

M. Moulé called attention to the frequency of the disease in fowls. The abdominal viscera, especially the liver, are affected ; these livers, mistaken for fatty livers, are selected for *pate de foie gras*, and one feeds on a veritable *puree* of bacilli.

MM. Morot, Villain, Thierry and Aurreggio recommended methods of examination of diseased cattle.

M. Thomassen (Utrecht), said in the Abattor of Amsterdam, five per cent. of the cattle are tuberculous. In hogs the per cent. is a little less.

M. Van Hertsen, (Brussels), Segin (Luxembourg), Robinson (Scotland), Degive, Peuch, Larmet and Guinard all added substantiation of the infectiousness of tuberculosis, and the necessity of proscription of the meat and milk of diseased animals, and indemnity to the owner.

By a large majority the following was adopted : "It is necessary to preserve by every means, including the indemnifying of those interested, the general application of the principle of seizure and total destruction of all meat coming from tuberculous animals, no matter what the gravity of the specific lesions in the animal."

Meeting July 27th, M. Villemin presiding. M. de Brun, (Beyreuth), read a paper on Tuberculosis in Syria.

M. Arloing presents in the name of M. Cartier, these conclusions :

1st. To patients to whom the use of raw meat seems necessary, the raw meat of the sheep or goat should be recommended.

2d. To those who desire to drink the blood at the abattoirs, counsel the blood of the goat or sheep.

3d. The blood of the goat or sheep should likewise be used for clarifying wine or other fluids.

The meetings July 28th, were occupied by some twenty-seven communications upon the heredity of tuberculosis, the presence of the disease in Asia Minor and Chili, its localization in udder, salivary glands, bone joints, etc.

On July 30th was discussed the "Means of introduction and propagation of tuberculosis virus in the economy ; and prophylactic measures."

On July 31st the question of early diagnosis of tuberculosis in men and animals was taken up. In conclusion the following additional resolutions were adopted :

1st. "There should be placed in the power of the Boards of Health (Conseils d'Hygiéne) all questions relative to the contagious diseases of the domestic animals, including those which do not seem at present transmissable to man. To vaccina, glanders, hydrophobia, anthrax and tuberculosis can in effect be added, later, other infectious diseases, requiring an equally common protection."

2d. The resolution of the second meeting.

3d. There should be edited simple instructions which should be profusely spread in village and country, and in which should be indicated the means to employ to shelter from the danger of tuberculous infection by alimentation, particularly with milk, and to destroy the virulent germs contained in the sputa of tuberculosis patients.

4th. There should be submitted to a special surveillance the dairies devoted to the industrial production of milk, to assure that the cows are not affected with contagious disease susceptible of being communicated to man.

The Assembly, by acclamation, name M. Villemin as president of the next Congress.

The next reunion is fixed in two years.

The present Committee is charged with the organization of the Congress of 1890. H.

---

Observations on the Boa Constrictor.—This snake was purchased from a dealer May 20, 1887, and at that date had been in stock four months without eating. After arrival at the zöological gardens it remained in a sluggish condition until January 31. 1888, when it fed, having lived without food for over year. Since then the snake has devoured seventeen pigeons.

C.

---

Notes.—We take pleasure in informing our readers that in the January number of the Journal will be published a monograph, with twenty-nine illustrations on rickets in monkeys, lions, bears and birds, by Mr. J. Bland Sutton, F. R. C. S., London. This will be the most complete work on rickets in animals, that has yet been published, as it is an embodiment of Mr. Bland's latest research in the matter. In the same number will be an article on horned mammals, with some observations on policerate, or multiple horned sheep, by Dr. R. W. Shufeldt, F. Z. S. C.

